# Prediction of fluid oil and gas volumes of shales with a deep learning model and its application to the Bakken and Marcellus shales

**DOI:** 10.1038/s41598-022-23406-3

**Published:** 2022-12-02

**Authors:** Şamil Şen

**Affiliations:** 1 Istanbul University-Cerrahpaşa, Istanbul, Turkey; 2 Shalesys Energy, Houston, TX USA

**Keywords:** Solid Earth sciences, Energy science and technology

## Abstract

The fluid oil and gas volumes (S1) retained within the shales are one of the most important parameter of producible fluid oil and gas saturations of shales together with total organic carbon content. The S1 volumes can directly be obtained by Rock-Eval pyrolysis analysis. However, it is time consuming and not practical to obtain samples from all intervals of all wells in any shale play. S1 volumes prediction with a deep learning (DL) model have increasingly became important with the booming exploration and development of shale oil and gas resources. S1 volumes of shales are controlled by organic matter richness, type and maturity together with reservoir quality and adsorption capacity which are mainly effected by age, depth, organic content, maturity and mineralogy. A dataset consisting of 331 samples from 19 wells of various locations of the world-class organic-rich shales of the Niobrara, Eagle Ford, Barnett, Haynesville, Woodford, Vaca Muerta and Dadaş has been used to determination of a DL model for S1 volumes prediction using Python 3 programing environment with Tensorflow and Keras open-source libraries. The DL model that contains 5 dense layers and, 1024, 512, 256, 128 and 128 neurons has been predicted S1 volumes of shales as high as R^2^ = 0.97 from the standard petroleum E&P activities. The DL model has also successfully been applied to S1 volumes prediction of the Bakken and Marcellus shales of the North America. The prediction of the S1 volumes show that the shales have lower to higher reservoir quality and, oil and gas production rate that are well-matches with former studies.

## Introduction

Over the past decade or so, two technologies (i.e., horizontal drilling coupled with large-scale, multistage hydraulic fracturing) have made it possible to extract hydrocarbons trapped in self-sourced shale plays^1,2^. The development that knowns as shale revolution has moved to US world leadership on oil and gas production. A workflow for de-risking productivity of an unconventional shale play is in part based on our examination of the S1 X 100/TOC ratio, which represent potentially producible fluid oil and gas saturations. Thus, the S1 is one of the most important parameters of producible fluid oil and gas saturation of shales together with total organic carbon content (TOC). Although minimum 100 mg oil/g TOC threshold were offered to the producible fluid oil and gas saturations of shales^3–6^, recent studies have considered that the threshold ranges from more than 100 mg oil/g TOC at the onset of oil expulsion to less than 40 mg oil/g TOC at the end of expulsion depend primarily on maturity^7–9^. The S1 is referred to as the available “free”^10^ or, more correctly, “volatile” fluid hydrocarbons volumes^7,9^ retained within the source rocks. The S1 volumes can be obtained by Rock-Eval pyrolysis analysis. However, it is time consuming and not practical to obtain samples from all intervals of all wells in any shale play. Organic matter richness, type, maturity and, reservoir quality and adsorption capacity control S1 volumes of shales. Reservoir quality and adsorption capacity of shales are mainly controlled by age, depth and mineralogy together also with organic matter richness and maturity^7–9,11–18^. On the one hand, oil and gas occurrences which are important factors of S1 volumes are related with time–temperature index which is controlled by burial history of the shales. On the other hand, age and depth affect reservoir quality of shales because old and deeply buried shales have principally less porosity and permeability. Mineral contents of the shales cause to increase or decrease of S1 volumes depend on their reservoir qualities and adsorption capacities. S1 volumes can be effected by contamination and fracture zones^14,15,17,18^. It is important to note that S1 volumes is more vulnerable to loss of light oil due to evaporation, sample handling and, preparation before analysis. Although loss of S1 is often estimated to be 35%^19^, the correction of S1 for “evaporative loss” is an important step to restore the present-day values to original values. Oil API gravity^17,20^, which is partially controlled by maturity^7–9^, is a major control on evaporative loss from C15^+^ (lower boiling range point components). In this study, correction of S1 is not applied to the training and validation dataset, but it is applied to the predictions.

Although there are many machine learning studies^21–25^ to predict TOC values, only two machine learning studies^23,26^ has predicted S1 volumes, so far. In addition, the two predictions are low accuracy (R^2^ = 0.78 and 0.91) because they used small number of well logs and S1 datasets taken from Goldwyer Shale of the Canning Basin of Australia and the Shahejie Shale of the Bohai Bay Basin of Chine. While the study of Johnson et al.^23^ used Levenberg–Marquardt training algorithm with Matlab programing environment with the established relationships between the 6 well logs and laboratory measured 96 S1 data, the study of Wang et al.^26^ used both back propagation artificial neural network (BPANN) and convolutional neural network (CNN) model with the established relationships between the one well log and laboratory measured 125 S1 data. Although Wang et al.^26^ study has effective tools such as both BPANN and CNN, its prediction is low accuracy probably because any log data presents maturity which is very important to S1 volumes prediction^7,9^ and one well log and laboratory measured 125 S1 data is very low. Aim of this study is to determine a deep learning model for S1 volumes prediction based on data of world-class shales and to apply for the Bakken and Marcellus shales using Python 3 programing environment with Tensorflow and Keras open-source libraries.

## Training and validation dataset

**Figure 1:**
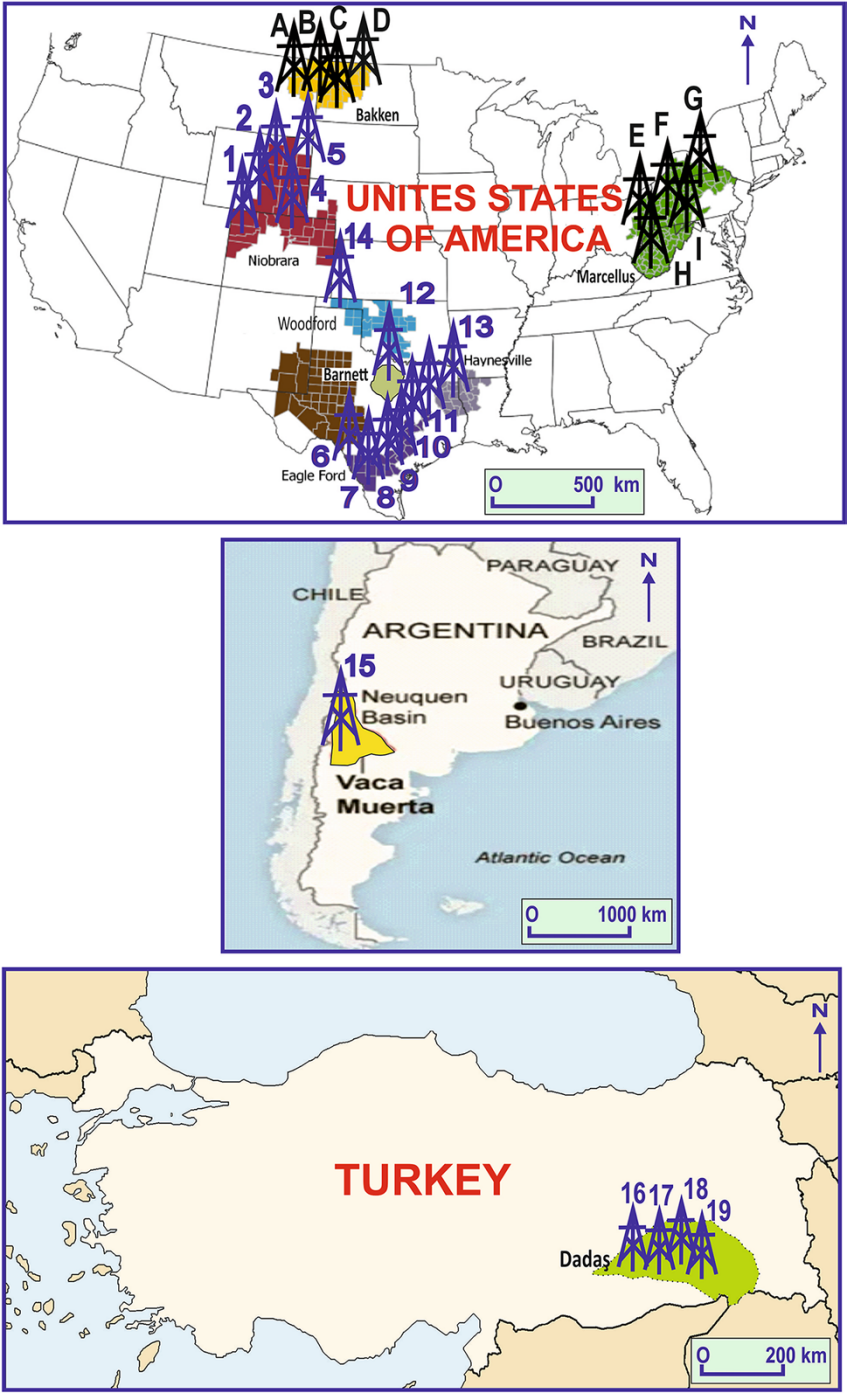
Location map of the wells of the training and validation dataset (1–19) and the S1 volumes prediction dataset (A–I). 1–5 = Niobrara Shale, 6–11 = Eagle Ford Shale, 12 = Barnett Shale, 13 = Haynesville Shale, 14 = Woodford Shale, 15 = Vaca Muerta Shale, 16–19 = Dadaş Shale, A, B, C, D = Bakken Shale, E, F, GHI = Marcellus Shale. This figure was generated with modification of public EIA maps (www.eia.gov) using CoralDRAW software.

The dataset consists of 331 instances and 8 variables from the 19 wells of the well-known shales of the world (Fig. 1, Table 1, Datashare 1). The variables that contain S1, age, depth, TOC%, Requ%, quartz%, clay%, carbonate% are selected because organic matter richness, type and maturity control generated oil and gas volumes and, age, depth and mineralogy control reservoir quality and adsorption capacities of shales that impact oil and gas accumulations. Note that all shale samples of the dataset are selected from Type II organic matter that capable to produce oil and oil cracking secondary gas depending on maturity. Thus, type has not included to the variables and this study is related with only II organic matters model and predictions.

**Table 1:** Training and validation dataset summary.

	S1_mg_oil/g_TOC	Age_BA	Present_Depth_km	TOC_%	Requ_%	Quartz_%	Clay_%	Carbonate_%
**Dataset summary**								
Count	1324.000000	1324.000000	1324.000000	1324.000000	1324.000000	1324.000000	1324.000000	1324.000000
Mean	2.910211	0.243353	2.339335	3.977613	0.899849	23.889758	30.663867	33.934169
Std	2.133312	0.143739	0.992484	2.036037	0.200838	11.846613	15.686748	25.604037
Min	0.100000	0.090000	1.110000	0.800000	0.580000	1.440000	0.000000	0.000000
25%	1.370000	0.090000	1.170000	2.540000	0.780000	15.720000	16.480000	10.800000
50%	2.440000	0.340000	2.160000	3.590000	0.840000	22.900000	31.000000	27.000000
75%	3.790000	0.360000	3.110000	5.200000	0.960000	32.460000	40.000000	56.540000
Max	11.250000	0.440000	4.870000	11.800000	1.600000	53.330000	71.300000	95.160000

The variables are world-class productive shales of the Niobrara (1, 2, 3, 4, 5 wells^27^), Eagle Ford (B, C, D, X wells^28^ and K1, K2 wells^29^), Barnett (Mesquite well^14^), Haynesville (SS-2 well^30^), Woodford (H2 well^31^), Vaca Muerta (1010 well^32^) and, Dadaş (Doğan 1, Soğuktepe 1, K. Migo 2 wells^8^ and Calıktepe 2 well^33^). It is important to note that the dataset variables and instances are standard E&P activities of the petroleum sector.

The TOC and S1 values of the dataset were obtained from Leco analyzer or Rock-Eval pyrolysis. Maturity calculated from Rock-Eval pyrolysis maximum temperature (Tmax) as equivalent vitrinite reflectance (Requ). Mineralogy of the dataset was measured with whole rock XRD.

## DNN model, training and validation

**Figure 2:**
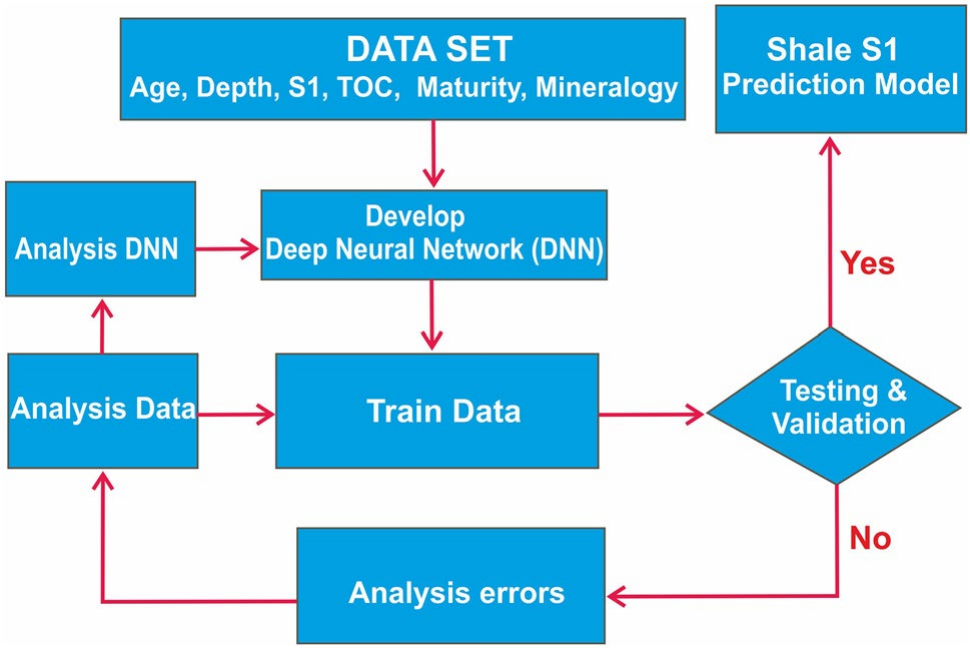
A proposed workflow showing the steps followed in this study to predict the S1 volumes of shales.

The dataset has been uploaded to the Jupyter Notebook that supports Python 3 programing environment. The DL workflow for S1 volumes prediction is given in the Fig. 2. The dataset shape contains 8 variables and 331 instances from 19 wells of the well-known shales. It is important to note that pretreatment methods have been applied to the dataset. First, instances that TOC values lower than 1% has been deleted from dataset due to absence any fluid oil and gas volumes potential. Second, the dataset has been increased to 1324 instances with data augmentation that is giving opportunity to generating new data points from existing data due to overfitting or simple learning solutions (Fig. 1, Table 1, Datashare 1). The input data is normalized to eliminate bias toward variables having larger values^34^. The standard normalized method has been selected in this study because lower loss index and more accuracy was obtained by the method during training and validation runs. The method known as Z-score and an element (x) is calculated using an arithmetic mean (μ) and standard deviation (σ), according to the following Eq. (1):

1 $$ {\text{z}} = \left( {{\text{x}}{-}\upmu } \right)/\upsigma . $$

A multilayer perceptron method is selected in this study. The method represents most basic deep neural network (DNN) which is composed of a series of fully connected layers. The method has the advantage of learning non-linear models, provides quick predictions after training and the same accuracy ratio with even with smaller samples.

A DNN model which contains 5 dense layers and, 1024, 512, 256, 128 and 128 neurons have been used for shale S1 volumes prediction because lower loss index and more accuracy was obtained by the dense layers and neurons during training and validation runs. The dataset has been spilled as 80% training and 20% validation. The training and validation instances use to find the best representation model of the dataset. This comparison is done by plotting calculated versus measured S1 volumes of the testing instances, and the quality of the model is measured by a best-fit linear line between the two S1 volumes. For a perfect fit of the data, a slope of one is required. The R^2^ is also provided so that how the differences of one variable can be explained by the other variable can be analyzed or the quality of the fit can be assessed.

The training objective is to find a Deep Neural Network (DNN) function with a minimum value of loss index. Mean Squared Error method was used to calculate loss index. Error minimization has been done by fine tuning of increasing the complexity of the neural network by adding more neurons and layers due to obtain low final training and validation losses as well as a high coefficient of determination (R^2^). The lines connecting the nodes are called activation functions, which are used to transform the activation level of a neuron into an output^35^. For a neuron j in this fully connected neural networks, its output yj is calculated by Eq. (2)

2 $$ {\text{yj}} = {\text{F}}\left( {\sum {\text{i}}\;{\text{wij}}\;{\text{xi}} + {\text{bj}}} \right) $$

where xi denotes the output of neuron i at the previous layer; wij and bj are the weight and bias, respectively; and F represents the activation function. Summarization of all neurons at a layer can thus be written as Eq. (3)

3 $$ {\text{y}} = {\text{F}}\left( {{\text{wx}} + {\text{b}}} \right). $$

During training, weights and biases were adjusted to minimize a loss function. The model was optimized by an Adam optimizer^36^ with a loss function of MSE. F was set as a rectified linear unit (ReLU) activation function.

## Prediction model and its applications

**Figure 3:**
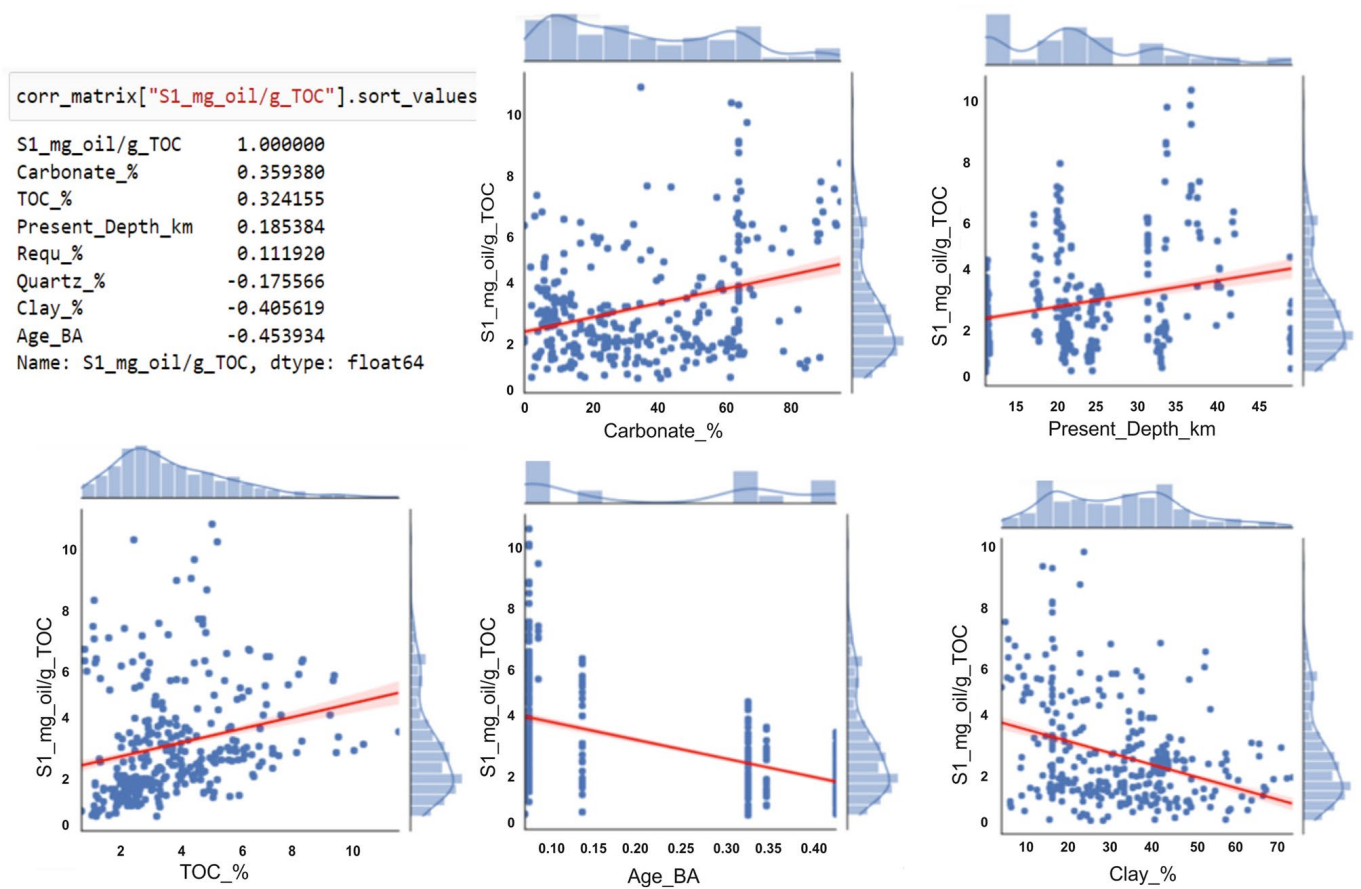
S1 correlation plots of the inputs and target of the training and validation dataset.

**Figure 4:**
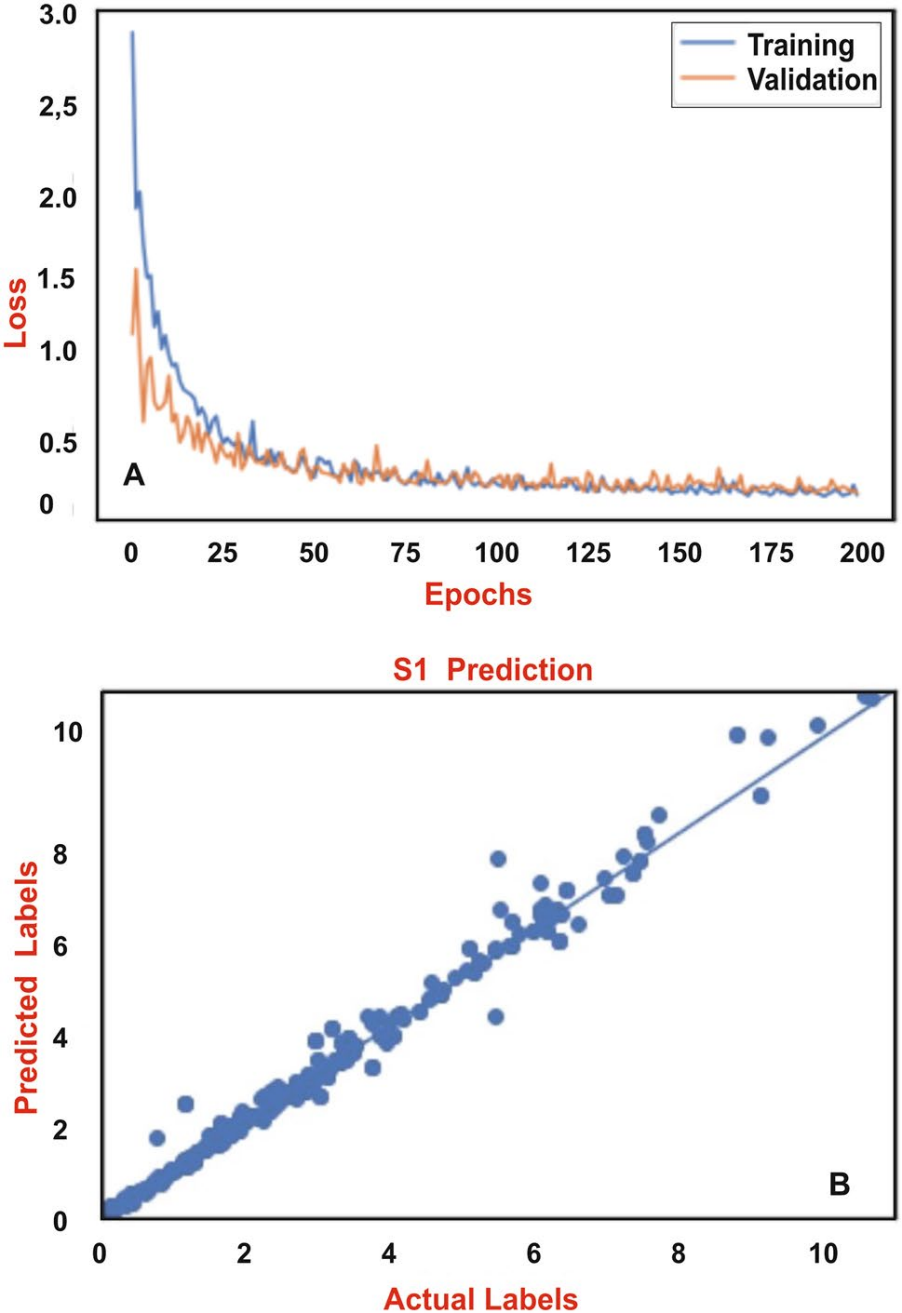
(**A**) The training and validation loss plot with 200 epochs, (**B**) Linear correlation coefficient plot of the real and predicted S1. R^2^ is as high as 0.97.

Plots of correlations show that carbonate content, TOC, present depth and maturity cause increase in S1 volumes, whereas age, quartz content and clay content cause decrease in S1 volumes (Fig. 3). Best Mean Squared Error history was obtained using 1024, 512, 256, 128 and 128 neurons and 5 dense layers (Table 2, Fig. 4A). Linear correlation coefficient using the DNN parameters has predicted as high as R^2^ = 0.97 (Fig. 4B). The DL model has been applied to S1 volumes prediction of the Bakken and Marcellus shales.

**Table 2:** The deep neural network (DNN) that includes 7 inputs, 1 target, and 1024, 512, 256, 128, 128 neurons with 5 layers.

Layer (type)	Output shape	Param #
**Model: “functional_1”**		
Input_1 (InputLayer)	[(None, 7)]	0
Dense (Dense)	(None, 1024)	8192
Dense_1 (Dense)	(None, 512)	524,800
Dense_2 (Dense)	(None, 256)	131,328
Dense_4 (Dense)	(None, 128)	32,896
Dense_5 (Dense)	(None, 1)	129

Total params: 697,345.

Trainable params: 697,345.

Non-trainable params: 0

### Prediction of fluid oil volumes of the Bakken shale

The Bakken Formation is an unconventional play which locates North America (Fig. 1). This formation is divided into three members. The Upper Bakken, the Middle Bakken, and the Lower Bakken. The Upper and Lower Bakken members mainly consists of dark-gray to brownish-black to black, fissile, slightly calcareous, organic-rich shale, which was deposited in an offshore marine environment. The DL model has been applied to the 4 wells^37,38^ (A, B, C, D wells in the Fig. 1). The Upper and Lower Bakken shales are represented by beginning of late oil windows with average of 0.92% Requ in the wells. TOC contents range from 6.58 to 16.69 wt.% with an average of 11.44 wt.%. Quartz, clay and carbonate contents are 45.75%, 42.09% and 1.10%, respectively. Predicted S1 volumes of the Bakken Shales vary from 1.3 to 7.6 mg oil/g TOC with an average of 3.64 mg oil/g TOC based on the DL model. In addition, average of evaporation loss corrected S1 volumes with 40 API gravity is 4.86 mg oil/g TOC. Thus, the average oil saturation index (OSI) of the Upper and Lower Bakken Shales has been calculated as 42.83 mg oil/g TOC (Table 3 and Datashare 1). The data show that the shales have lower reservoir quality and lower oil production rate because the OSI is very close to threshold accepting as 40 mg oil/g TOC^7–9^, or lower than accepting as 100 mg oil/g TOC^6^. The finding well-matches with former studies^6,39^ on the Bakken Shales.

**Table 3:** Prediction dataset of the Bakken and Marcellus shales and, predicted S1 volumes, evaporation loss corrected S1 volumes and OSI based on the DL model.

Well	Age_BA	Depth_km	TOC_%	Requ_%	Quartz_%	Clay_%	Carbonate_%	Predicted S1	Corrected S1	OSI
Marcellus_1	0.39	1.53	2.30	1.00	26.80	42.40	19.70	2.9845467	7.22	314.03
Marcellus_1	0.39	1.53	5.10	1.00	24.80	52.40	2.60	3.8604107	9.34	183.18
Marcellus_1	0.39	1.54	6.00	1.00	22.80	41.90	12.00	3.3182776	8.03	133.84
Marcellus_1	0.39	1.54	3.20	1.00	27.90	49.80	4.40	3.195042	7.73	241.63
Marcellus_1	0.39	1.54	3.80	1.00	30.10	48.90	2.90	3.0421793	7.36	193.74
Marcellus_1	0.39	1.54	8.10	1.00	28.50	38.00	4.90	2.5373392	6.14	75.81
Marcellus_1	0.39	1.54	10.20	1.00	30.70	34.10	2.40	2.5453644	6.16	60.39
Marcellus_1	0.39	1.55	12.80	1.00	23.10	31.00	6.20	2.5044882	6.06	47.35
Marcellus_1	0.39	1.55	13.60	1.00	19.60	30.30	10.60	3.4281745	8.30	61.00
Marcellus_2	0.39	2.82	3.40	2.00	33.60	44.90	3.40	3.0143514	10.31	303.21
Marcellus_2	0.39	2.82	3.10	2.00	35.40	40.70	8.10	1.67386	5.72	184.66
Marcellus_2	0.39	2.83	6.40	2.00	32.70	39.90	3.60	5.3681893	18.36	286.86
Marcellus_2	0.39	2.83	8.30	2.10	30.90	35.20	7.40	3.7243223	12.74	153.46
Marcellus_2	0.39	2.84	10.70	2.10	44.40	22.30	6.00	4.776375	16.34	152.67
Marcellus_3	0.39	2.17	4.05	1.36	31.0	65.0	4.0	2.7303286	9.34	230.56
Marcellus_3	0.39	2.18	3.33	1.36	37.0	60.0	3.0	1.5210965	5.20	156.22
Marcellus_3	0.39	2.18	2.31	1.36	36.0	63.0	1.0	1.453183	4.97	215.15
Marcellus_3	0.39	2.18	4.28	1.36	32.0	49.0	19.0	2.315579	7.92	185.03
Marcellus_3	0.39	2.18	4.53	1.37	32.0	57.0	11.0	2.411799	8.25	182.08
Marcellus_3	0.39	2.20	6.22	1.37	42.0	56.0	2.0	3.8097692	13.03	209.48
Marcellus_3	0.39	2.20	6.77	1.37	44.0	33.0	23.0	1.3706208	4.69	69.24
Marcellus_4	0.39	2.33	1.94	1.37	36.0	64.0	1.0	1.5719676	5.38	277.12
Marcellus_4	0.39	2.34	3.02	1.37	30.0	69.0	1.0	1.6019534	5.48	181.41
Marcellus_4	0.39	2.35	2.64	1.37	32.0	67.0	1.0	1.5634888	5.35	202.54
Marcellus_4	0.39	2.35	5.66	1.37	60.0	38.0	3.0	3.776731	12.92	228.21
Marcellus_4	0.39	2.36	6.83	1.38	50.0	44.0	6.0	4.966872	16.99	248.71
Marcellus_4	0.39	2.36	4.81	1.40	38.0	60.0	2.0	2.6137424	8.94	185.84
Marcellus_4	0.39	2.36	4.13	1.41	45.0	46.0	8.0	1.2467439	4.26	103.24
Marcellus_5	0.39	2.15	2.15	2.67	26.0	71.0	3.0	0.69902396	2.39	111.19
Marcellus_5	0.39	2.16	2.71	2.67	26.0	66.0	8.0	1.2882397	4.41	162.57
Marcellus_5	0.39	2.16	2.67	2.67	28.0	69.0	3.0	0.7913606	2.71	101.37
Marcellus_5	0.39	2.16	2.96	2.67	44.0	54.0	2.0	0.80809015	2.76	93.37
Marcellus_5	0.39	2.16	3.10	2.67	39.0	52.0	8.0	1.4333402	4.90	158.13
Marcellus_5	0.39	2.16	7.28	2.68	36.0	60.0	4.0	2.8478527	9.74	133.79
Marcellus_5	0.39	2.17	3.07	2.68	38.0	53.0	9.0	1.4615773	5.00	162.82
Marcellus_5	0.39	2.17	2.81	2.68	36.0	60.0	5.0	1.0313363	3.53	125.52
**Average**	**0.39**	**2.14**	**5.12**	**1.66**	**34.15**	**50.22**	**6.14**	**2.480211575**	**7.72**	**169.87**
Well_1_Upper_Bakken	0.35	3.17	13.57	0.94	39.57	44.87	1.60	5.9196873	10.66	78.52
Well_1_Upper_Bakken	0.35	3.18	7.41	0.94	45.08	42.29	1.34	2.0221586	2.59	34.93
Well_1_Upper_Bakken	0.35	3.18	9.14	0.94	48.90	36.24	0.60	1.3083813	1.67	18.32
Well_1_Upper_Bakken	0.35	3.18	13.33	0.94	63.59	24.07	0.90	3.3798652	4.33	32.45
Well_1_Lower_Bakken	0.35	3.21	6.58	0.94	38.36	49.23	1.50	2.4263053	3.11	47.20
Well_1_Lower_Bakken	0.35	3.22	11.34	0.94	38.04	53.92	1.00	4.4843583	5.74	50.62
Well_1_Lower_Bakken	0.35	3.22	15.86	0.94	50.27	36.63	1.20	4.5060062	5.77	36.37
Well_1_Lower_Bakken	0.35	3.22	8.59	0.94	38.44	50.59	0.15	1.8996017	2.43	28.31
Well_2_Upper_Bakken	0.35	3.27	13.57	0.94	39.57	44.87	1.60	5.3731914	6.88	50.68
Well_2_Upper_Bakken	0.35	3.27	7.41	0.94	45.08	42.29	1.34	1.9284317	2.47	33.31
Well_3_Upper_Bakken	0.35	2.56	12.43	0.86	53.24	35.41	0.15	3.0873027	3.95	31.79
Well_3_Upper_Bakken	0.35	2.56	13.33	0.93	63.59	24.07	1.00	2.624426	3.36	25.20
Well_4_Lower_Bakken	0.35	3.01	6.58	0.82	38.36	49.23	1.50	3.9669528	5.08	77.17
Well_4_Lower_Bakken	0.35	3.01	11.34	0.85	38.04	53.92	1.00	6.004355	7.69	67.77
Well_4_Lower_Bakken	0.35	3.01	16.69	0.95	41.55	49.22	1.40	7.6387672	9.78	58.58
Well_4_Lower_Bakken	0.35	3.02	15.86	0.95	50.27	36.63	1.30	1.7363921	2.22	14.01
**Average**	**0.35**	**3.08**	**11.44**	**0.92**	**45.75**	**42.09**	**1.10**	**3.644136425**	**4.86**	**42.83**

TOC, Total organic carbon content; Requ, Vitrinite reflectance equivalent; S1, Fluid oil and gas volumes as mg HC/g TOC; OSI, Oil saturation index as mg oil/g TOC.

### Prediction of fluid oil and gas volumes of the Marcellus shale

The Middle Devonian Marcellus Formation is a distal marine shale within a foreland succession deposited in the Appalachian Basin (Fig. 1). The Marcellus Shale has attracted great attention as an important gas-producing shale of US. The DL model has been applied to the 5 wells^40,41^ of the Marcellus Shale (E, F, G, H, I wells in the Fig. 1). The shale is represented by late oil (well 1), condensate-wet gas (well 3 and 4) and dry gas (well 5) windows because Requ of the wells are 1%, 1.36–1.38% and 2.67–2.68%, respectively. TOC contents range from 1.94 to 13.6 wt.% with an average of 5.12 wt.%. Quartz, clay and carbonate contents are 34.15%, 50.22% and 6.14, respectively. Predicted S1 volumes of the Marcellus Shale vary from 0.69 to 5.36 mg oil/g TOC with an average 2.48 mg oil/g TOC based on the DL model. In addition, average of evaporation loss corrected S1 volume with 45–50 API gravities is 7.72 mg oil/g TOC. Thus, the average oil saturation index (OSI) of the Marcellus Shale has been calculated as 169.87 mg oil/g TOC (Table 3 and Datashare 1). The data show that the Marcellus Shale has higher reservoir quality and higher oil and gas production rate because the OSI is higher than threshold both accepting as 40 mg oil/g TOC^7–9^ and accepting as 100 mg oil/g TOC^6^. The finding also well-matches with former studies^42,43^ on the Marcellus Shale.

## Conclusions

The S1 or fluid oil and gas volumes retained within the shales can be obtained by Rock-Eval pyrolysis analysis. Organic matter richness, type, maturity, age, depth, and mineralogy control S1 volumes. Pyrolysis analysis is time consuming and S1 volumes are more vulnerable to loss of light oil. Thus, this study has determined a deep learning model for S1 volumes prediction and is applied to the Bakken and Marcellus shales using Python 3 programing language, Tensorflow and Keras libraries. The dataset contains 331 instances and 8 variables from the 19 wells of the world-class organic-rich shales of the Niobrara, Eagle Ford, Barnett, Haynesville, Woodford, Vaca Muerta and Dadaş. Best Mean Squared Error loss history to S1 volumes prediction has obtained using 1024, 512, 256, 128 and 128 neurons and 5 layers. Linear correlation coefficient using the DNN parameters was predicted as high as R^2^ = 0.97. The correlation shows that S1 volumes of the organic rich shale formations could quickly be predicted from the DL model obtained by dataset of standard E&P activities of the petroleum sector. The DL model has successfully been applied to S1 volumes prediction of the Marcellus and Bakken shales. Average predicted S1 volumes of the Bakken and Marcellus shales based on the DL model are 3.64 mg oil/g TOC and 2.48 mg oil/g TOC, respectively. Average evaporation loss corrected S1 values are 4.86 mg oil/g TOC in the Bakken Shales and 7.72 mg HC/g TOC in the Marcellus Shale. The predictions show that the Bakken and Marcellus shales have lower to higher reservoir quality and, oil and gas production rate that are well matched with former studies, respectively.

## Methods

### Supervised deep learning

Supervised Deep Learning (DL) method has been used to predict of the S1 volumes of the shales based on actual training and testing data of laboratory analysis of shale samples. The DL method is the most important technique for machine learning and artificial intelligence that uses multilayered neural networks to extract high-order features^44,45^. Deep Neural Network (DNN) is mathematical models based on the neural structure of intelligent organisms, specifically the human brain^46–48^. Their main characteristics are learning, generalization and abstraction capacities, which they obtain through the search of relationships, automatic construction of models and corrections based on experience, in order to reduce their own errors. Multilayer perceptron is the most commonly used neural network and it is structured in a way that the number of input features is the same as the number of neurons in the input layer. The output layer has the same number of neurons as the classes of interest. Hidden layers in between and the neurons in each one of these layers may vary according to the problem characteristics^49–52^.

### Shale analyses

TOC and S1 volumes of the dataset were obtained from Leco analyzer and/or Rock-Eval 6 pyrolysis. Rock-Eval data analyses were carried out by Institut Français du Petrole standards. The results of the analysis were used to calculate the S1 (mg HC/g rock) liberated at temperatures under 300 °C. The samples were analyzed using the standard procedures^10^.

Equivalent vitrinite reflectance (Requ%) has been used as a thermal stress indicator in this study. Thus, the Eq. (4)^53^ was applied to the samples.

4 $$ {\text{Requ}}\% = 0.0{165} \cdot {\text{Tmax -}}\;{65},{143} $$

Quartz, clay and carbonate contents of the dataset were carried out with whole rock XRD analysis. Note that quartz, clay and carbonate contents of the Eagle Ford B, C, D, X wells are average values of K1 and K2 wells. Following XRD analyses, qualitative and quantitative determinations of mineral compositions have been calculated by Jade 7.0 Software, Inorganic Crystal Structure Database of the International Center for Diffraction Data, and the Reference Intensity Ratios of the Easy Quant Program.

Age and depth of the shales have been taken from given references in the dataset sections.

### Evaporation loss correction for S1

Evaporative loss from C15^+^ was calculated by below Eqs. (5, 6)^17^

5 $$ \% {\text{C15}}^{ + } {\text{loss}} = {\text{oil}}\;{\text{API}}\;{\text{gravity}}{-}\left( {{2}0,{799}/0.{412}} \right) $$

6 $$ {\text{S1cf}} = {1}/\left( {{1} - \% {\text{C15}}^{ + } {\text{loss}}} \right) $$

where S1 correlation factor is represented as S1cf.

API gravity for correction of S1 evaporate loss has been estimated with maturity-API gravity chart^13,17,54^.

## Supplementary Information

Supplementary Information.

## Data Availability

The data that support the findings of this study are available from Shalesys Energy Company. But restrictions apply to the availability of these data, which were used under license for the current study, and so are not publicly available. Data are however available from the authors upon reasonable request and with permission of Shalesys Energy Company.
