# Supplementary material for: Prediction of fluid oil and gas volumes of shales with a deep learning model and its application to the Bakken and Marcellus shales
Source: Sci Rep. 2022 Dec 2;12:20842. doi: 10.1038/s41598-022-23406-3 (PMC9718744; doi:10.1038/s41598-022-23406-3)
Supplement: Supplementary file 1 — Supplementary Information. [file 41598_2022_23406_MOESM1_ESM.pdf]

In [1]:

```
import pandas as pd
import numpy as np
import tensorflow as tf

import matplotlib.pyplot as plt
import seaborn as sns
%matplotlib inline
```

In [2]:

```
raw_dataset=pd.read_csv("S1V2.csv")
```

In [3]:

```
S1V2 = raw_dataset.copy()
S1V2.head()
```

Out[3]:

	Well	S1_mg_oil/g_TOC	Age_BA	Present_Depth_km	TOC_%	Requ_%	Quartz_%
0	Dadas_Dogan_1	3.08	0.44	2.49	10.60	0.70	33.7
1	Dadas_Dogan_1	3.53	0.44	2.52	11.80	0.73	22.5
2	Dadas_Dogan_1	3.34	0.44	2.54	9.74	0.71	15.8
3	Dadas_Dogan_1	2.12	0.44	2.57	4.50	0.81	17.0
4	Dadas_Dogan_1	2.63	0.44	2.60	4.20	0.63	47.4

In [4]:

```
S1V2.shape
```

Out[4]:

```
(1324, 9)
```

In [5]:

```
S1V2.describe()
```

Out[5]:

	S1_mg_oil/g_TOC	Age_BA	Present_Depth_km	TOC_%	Requ_%	Quartz_'
**count**	1324.000000	1324.000000	1324.000000	1324.000000	1324.000000	1324.00000
**mean**	2.910211	0.243353	2.339335	3.977613	0.899849	23.88975
**std**	2.133312	0.143739	0.992484	2.036037	0.200838	11.84661
**min**	0.100000	0.090000	1.110000	0.800000	0.580000	1.44000
**25%**	1.370000	0.090000	1.170000	2.540000	0.780000	15.72000
**50%**	2.440000	0.340000	2.160000	3.590000	0.840000	22.90000
**75%**	3.790000	0.360000	3.110000	5.200000	0.960000	32.46000
**max**	11.250000	0.440000	4.870000	11.800000	1.600000	53.33000

In [6]:

```
S1V2.info()
```

```
<class 'pandas.core.frame.DataFrame'>
RangeIndex: 1324 entries, 0 to 1323
Data columns (total 9 columns):
 #   Column            Non-Null Count  Dtype
---  ------            --------------  -----
 0   Well              1324 non-null   object
 1   S1_mg_oil/g_TOC   1324 non-null   float64
 2   Age_BA            1324 non-null   float64
 3   Present_Depth_km  1324 non-null   float64
 4   TOC_%             1324 non-null   float64
 5   Requ_%            1324 non-null   float64
 6   Quartz_%          1324 non-null   float64
 7   Clay_%            1324 non-null   float64
 8   Carbonate_%       1324 non-null   float64
dtypes: float64(8), object(1)
memory usage: 93.2+ KB
```

In [7]:

```
corr_matrix =S1V2.corr()
```

In [8]:

```
corr_matrix["S1_mg_oil/g_TOC"].sort_values(ascending=False)
```

Out[8]:

```
S1_mg_oil/g_TOC      1.000000
Carbonate_%          0.359380
TOC_%                0.324155
Present_Depth_km     0.185384
Requ_%               0.111920
Quartz_%            -0.175566
Clay_%              -0.405619
Age_BA              -0.453934
Name: S1_mg_oil/g_TOC, dtype: float64
```

In [9]:

```
sns.pairplot(S1V2)
```

Out[9]:

```
<seaborn.axisgrid.PairGrid at 0x17b4a42d310>
```

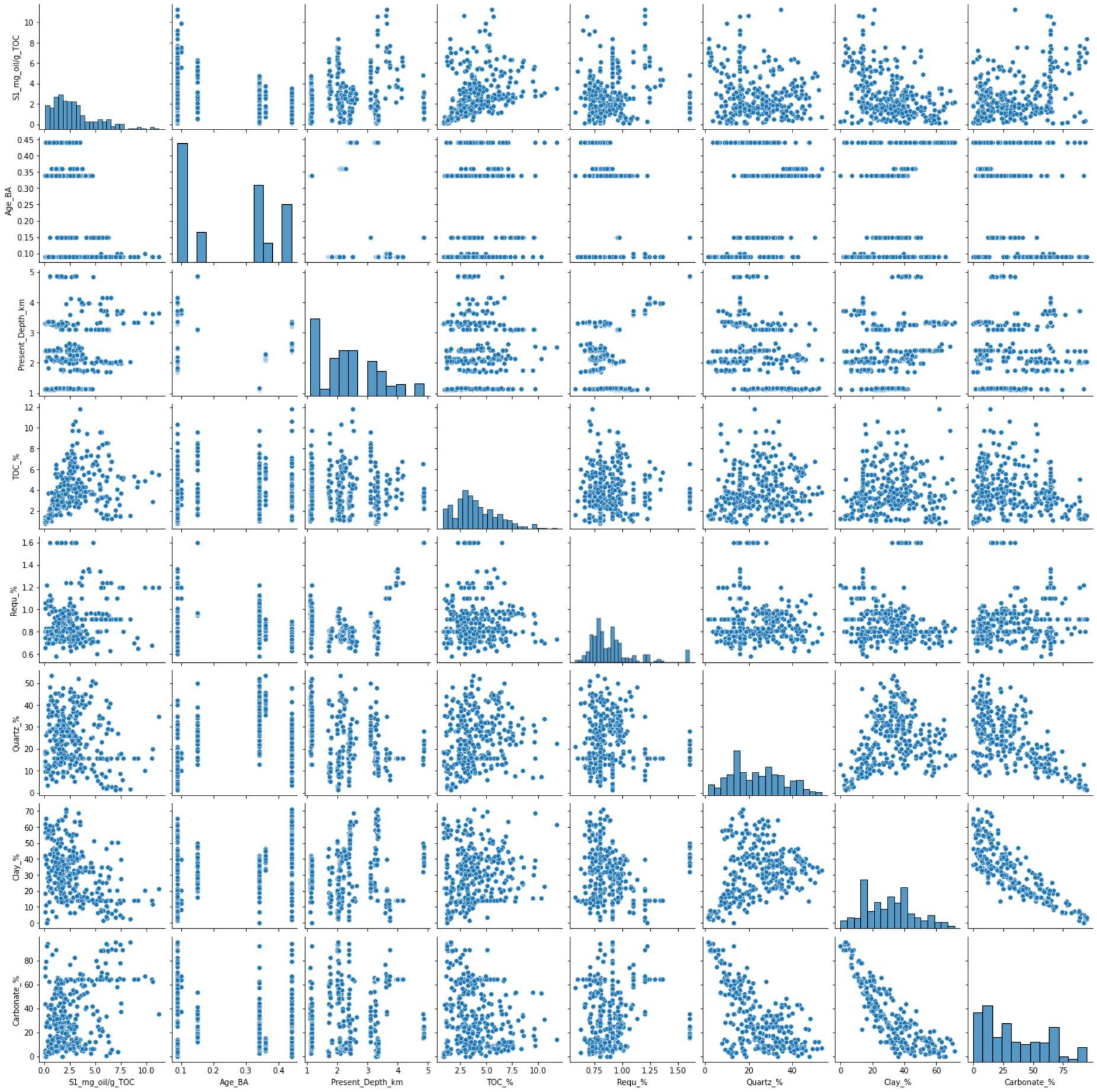

In [10]:

```
sns.set_theme(style="white")
plt.figure(figsize = (40,5), dpi = (100))
sns.jointplot(x = S1V2['Present_Depth_km'], y = S1V2['S1_mg_oil/g_TOC'], kind='reg', line_k
```

Out[10]:

```
<seaborn.axisgrid.JointGrid at 0x17b4a2c5940>
```

```
<Figure size 4000x500 with 0 Axes>
```

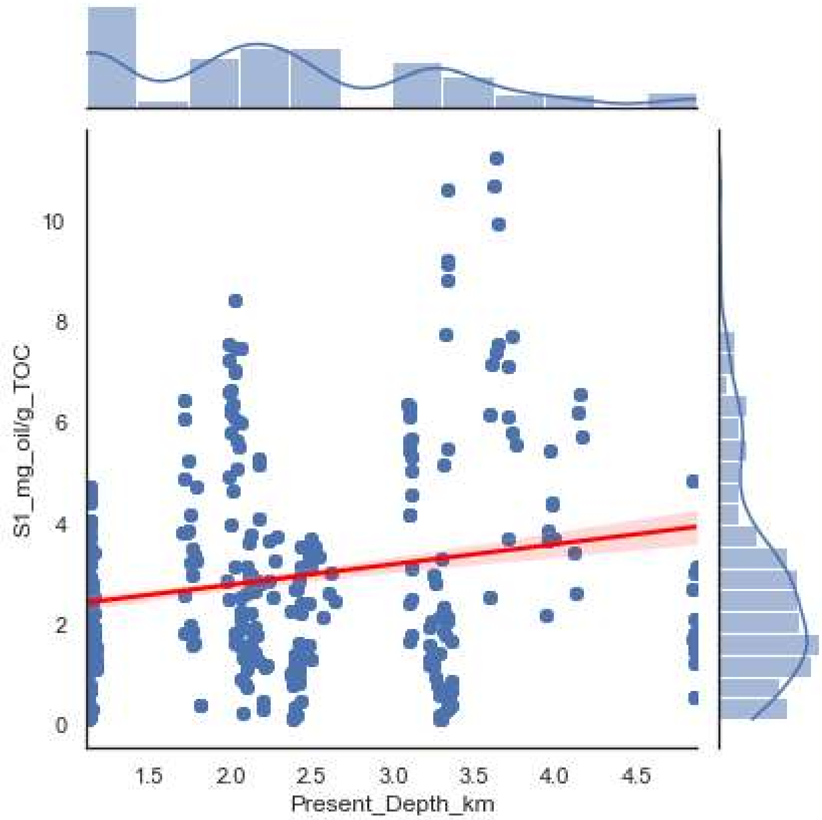

In [11]:

```
sns.set_theme(style="white")
plt.figure(figsize = (40,5), dpi = (100))
sns.jointplot(x = S1V2['TOC_%'], y = S1V2['S1_mg_oil/g_TOC'], kind='reg', line_kws={"color"
```

Out[11]:

```
<seaborn.axisgrid.JointGrid at 0x17b4e380b20>
```

```
<Figure size 4000x500 with 0 Axes>
```

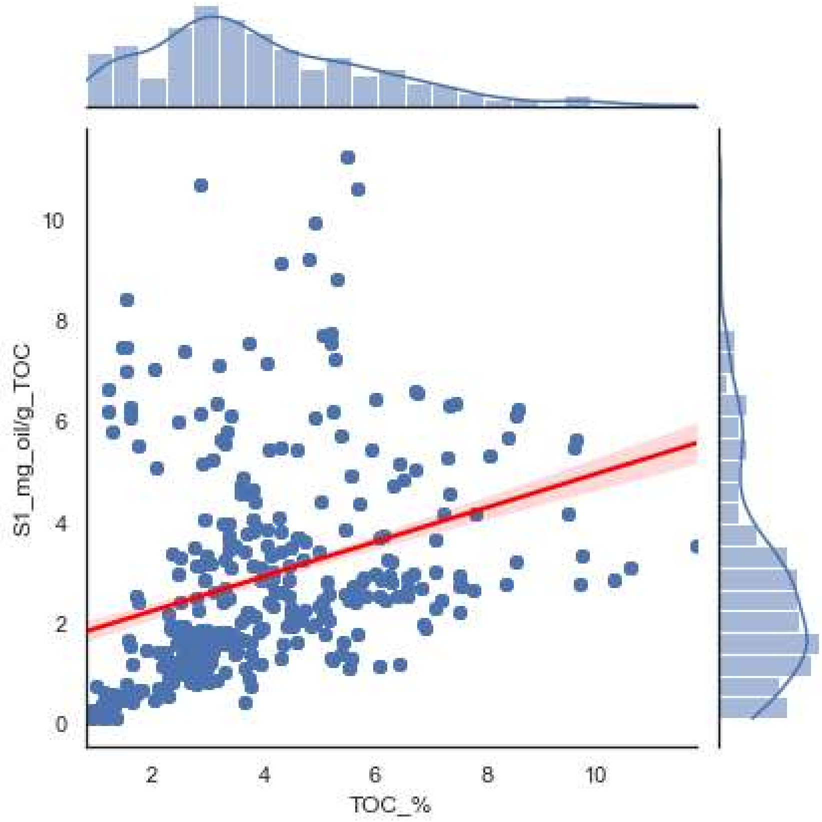

In [12]:

```
X = S1V2.iloc[:, 2:].values
y = S1V2.iloc[:, 1].values
```

In [13]:

```
X
```

Out[13]:

```
array([[ 0.44,  2.49, 10.6 , ..., 33.7 , 22.8 , 30.5 ],
       [ 0.44,  2.52, 11.8 , ..., 22.5 , 61.5 , 14.1 ],
       [ 0.44,  2.54,  9.74, ..., 15.8 , 68.6 , 13.9 ],
       ...,
       [ 0.36,  2.26,  5.83, ..., 42.63, 41.56,  6.  ],
       [ 0.36,  2.27,  6.22, ..., 41.39, 40.33, 11.  ],
       [ 0.36,  2.28,  6.14, ..., 42.04, 42.04,  6.  ]])
```

In [14]:

```
y
```

Out[14]:

```
array([3.08, 3.53, 3.34, ..., 2.52, 3.23, 3.74])
```

In [15]:

```
from sklearn.model_selection import train_test_split
X_train, X_test, y_train, y_test = train_test_split(X, y)
```

In [16]:

```
from sklearn.preprocessing import StandardScaler
sc = StandardScaler()
X_train = sc.fit_transform(X_train)
X_test = sc.transform(X_test)
```

In [17]:

```
X_train
```

Out[17]:

```
array([[-1.05128267, -0.28784462, -0.75511348, ..., -1.36831886,
        -1.08617345,  1.71072842],
       [ 0.68465536, -1.22754777, -1.17223165, ...,  1.2151847 ,
        -0.4909979 ,  0.27019623],
       [-1.05128267,  1.36163432, -0.28774012, ..., -0.68320949,
        -1.2490636 ,  1.23055102],
       ...,
       [ 0.68465536, -1.22754777, -1.17223165, ...,  1.2151847 ,
        -0.4909979 ,  0.27019623],
       [-0.63465754,  0.75182695,  1.93353973, ..., -0.1347845 ,
        -0.92954831,  0.73676536],
       [ 1.37903057,  0.07204169, -0.47368436, ...,  0.3123928 ,
         1.92102932, -1.18394423]])
```

In [18]:

```
from tensorflow.keras.layers import Input, Dense, Activation,Dropout
from tensorflow.keras.models import Model
```

In [19]:

```
input_layer = Input(shape=(X.shape[1],))
dense_layer_1 = Dense(1024, activation='relu')(input_layer)
dense_layer_2 = Dense(512, activation='relu')(dense_layer_1)
dense_layer_3 = Dense(256, activation='relu')(dense_layer_2)
dense_layer_4 = Dense(128, activation='relu')(dense_layer_3)
dense_layer_5 = Dense(128, activation='relu')(dense_layer_3)
output = Dense(1)(dense_layer_5)

model = Model(inputs=input_layer, outputs=output)
model.compile(loss="mean_squared_error" , optimizer="adam", metrics=["mean_squared_error"])
```

In [20]:

```
my_model = model
```

In [21]:

```
my_model.summary()
```

```
Model: "functional_1"
_________________________________________________________________
Layer (type)                 Output Shape              Param #   
=================================================================
input_1 (InputLayer)         [(None, 7)]               0         
_________________________________________________________________
dense (Dense)                (None, 1024)              8192      
_________________________________________________________________
dense_1 (Dense)              (None, 512)               524800    
_________________________________________________________________
dense_2 (Dense)              (None, 256)               131328    
_________________________________________________________________
dense_4 (Dense)              (None, 128)               32896     
_________________________________________________________________
dense_5 (Dense)              (None, 1)                 129       
=================================================================
Total params: 697,345
Trainable params: 697,345
Non-trainable params: 0
_________________________________________________________________
```

In [22]:

```
history = model.fit(X_train, y_train, batch_size=1, epochs=200, verbose=1, validation_split
```

```
Epoch 1/200
794/794 [==============================] - 8s 10ms/step - loss: 2.8813 - m
ean_squared_error: 2.8813 - val_loss: 1.0491 - val_mean_squared_error: 1.0
491
Epoch 2/200
794/794 [==============================] - 9s 12ms/step - loss: 1.8119 - m
ean_squared_error: 1.8119 - val_loss: 1.4443 - val_mean_squared_error: 1.4
443
Epoch 3/200
794/794 [==============================] - 13s 17ms/step - loss: 1.9127 -
mean_squared_error: 1.9127 - val_loss: 0.9473 - val_mean_squared_error: 0.
9473
Epoch 4/200
794/794 [==============================] - 15s 19ms/step - loss: 1.5916 -
mean_squared_error: 1.5916 - val_loss: 0.5142 - val_mean_squared_error: 0.
5142
Epoch 5/200
794/794 [==============================] - 15s 19ms/step - loss: 1.3913 -
mean_squared_error: 1.3913 - val_loss: 0.8587 - val_mean_squared_error: 0.
```

In [23]:

```
plt.plot(history.history["loss"])
plt.plot(history.history["val_loss"])
plt.xlabel("Epochs")
plt.ylabel("loss")
plt.legend(["Training","Validation"])
```

Out[23]:

```
<matplotlib.legend.Legend at 0x17b4f1b0e50>
```

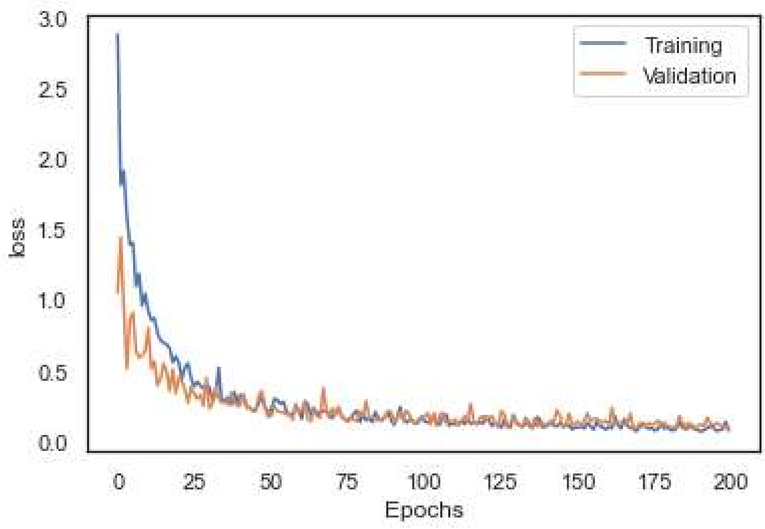

In [24]:

```
from sklearn.metrics import mean_squared_error
from math import sqrt
pred_train = model.predict(X_train)
print(np.sqrt(mean_squared_error(y_train,pred_train)))
pred = model.predict(X_test)
print(np.sqrt(mean_squared_error(y_test,pred)))
```

```
0.2544896026742563
0.3174872612227883
```

In [25]:

```
score = model.evaluate(X_test, y_test, verbose=1)
print("Test Score:", score[0])
print("Test Accuracy:", score[1])
```

```
11/11 [==============================] - 0s 33ms/step - loss: 0.1008 - mean_
squared_error: 0.1008
Test Score: 0.10079817473888397
Test Accuracy: 0.10079817473888397
```

In [26]:

```
predictions = model.predict(X_test)
np.set_printoptions(suppress=True)
print('Predicted labels: ', np.round(predictions)[:10])
print('Actual labels : ' ,y_test[:10])
```

```
Predicted labels:  [[5.]
 [4.]
 [0.]
 [1.]
 [3.]
 [1.]
 [4.]
 [4.]
 [3.]
 [4.]]
Actual labels :  [4.91 3.43 0.18 1.41 3.   1.46 3.32 3.73 3.08 5.45]
```

In [27]:

```
from sklearn.metrics import r2_score
y_true = np.round(predictions)
y_pred = y_test
r2_score(y_true, y_pred)
```

Out[27]:

```
0.9682033980996608
```

In [28]:

```
plt.scatter(y_test, predictions)
plt.xlabel('Actual Labels')
plt.ylabel('Predicted Labels]')
plt.title('Shale S1 Predictions')
lims = [0, 11]
plt.xlim(lims)
plt.ylim(lims)
_ = plt.plot(lims, lims)
```

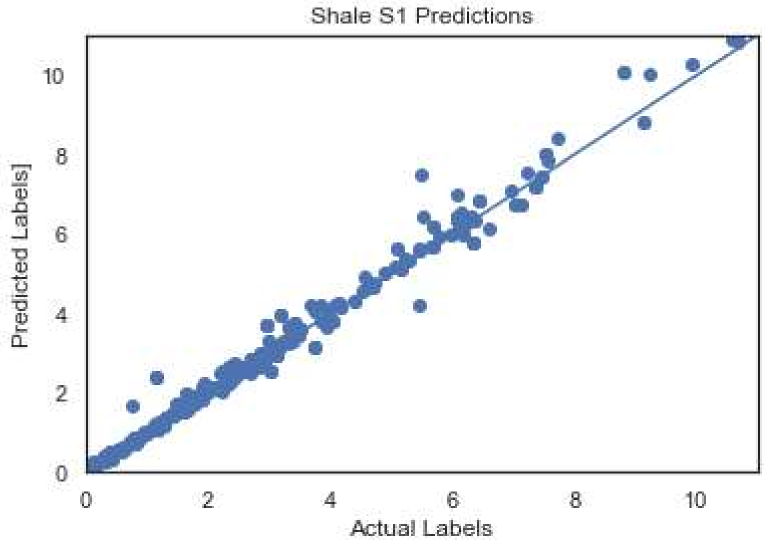

In [29]:

```
my_model.save('./saved_models/my_tf_model')
```

```
WARNING:tensorflow:From C:\Users\90532\anaconda3\lib\site-packages\tensorflo
w\python\training\tracking\tracking.py:111: Model.state_updates (from tensor
flow.python.keras.engine.training) is deprecated and will be removed in a fu
ture version.
Instructions for updating:
This property should not be used in TensorFlow 2.0, as updates are applied a
utomatically.
WARNING:tensorflow:From C:\Users\90532\anaconda3\lib\site-packages\tensorflo
w\python\training\tracking\tracking.py:111: Layer.updates (from tensorflow.p
ython.keras.engine.base_layer) is deprecated and will be removed in a future
version.
Instructions for updating:
This property should not be used in TensorFlow 2.0, as updates are applied a
utomatically.
INFO:tensorflow:Assets written to: ./saved_models/my_tf_model\assets
```

In [30]:

```
my_tf_saved_model = tf.keras.models.load_model(
 './saved_models/my_tf_model')
my_tf_saved_model.summary()
```

```
Model: "functional_1"
_________________________________________________________________
Layer (type)                 Output Shape              Param #
=================================================================
input_1 (InputLayer)         [(None, 7)]               0
_________________________________________________________________
dense (Dense)                (None, 1024)              8192
_________________________________________________________________
dense_1 (Dense)              (None, 512)               524800
_________________________________________________________________
dense_2 (Dense)              (None, 256)               131328
_________________________________________________________________
dense_4 (Dense)              (None, 128)               32896
_________________________________________________________________
dense_5 (Dense)              (None, 1)                 129
=================================================================
Total params: 697,345
Trainable params: 697,345
Non-trainable params: 0
_________________________________________________________________
```

In [1]:

```
from tensorflow.keras.models import save_model, load_model
import pandas as pd
```

In [2]:

```
model = load_model('./saved_models/my_tf_model',
 custom_objects=None,
 compile=True)
```

In [3]:

```
raw_dataset=pd.read_csv("MarcellusS1prediction.csv",sep=",")
```

In [4]:

```
MarcellusS1prediction= raw_dataset.copy()
MarcellusS1prediction.head()
```

Out[4]:

	Well	Age_BA	Present_Depth_km	TOC_%	Requ_%	Quartz_%	Clay_%	Carbonate_%
**0**	Marcellus_1	0.39	1.53	2.3	1.0	26.8	42.4	19.7
**1**	Marcellus_1	0.39	1.53	5.1	1.0	24.8	52.4	2.6
**2**	Marcellus_1	0.39	1.54	6.0	1.0	22.8	41.9	12.0
**3**	Marcellus_1	0.39	1.54	3.2	1.0	27.9	49.8	4.4
**4**	Marcellus_1	0.39	1.54	3.8	1.0	30.1	48.9	2.9

In [5]:

```
MarcellusS1prediction.info()
```

```
<class 'pandas.core.frame.DataFrame'>
RangeIndex: 36 entries, 0 to 35
Data columns (total 8 columns):
 #   Column            Non-Null Count  Dtype  
---  ------            --------------  -----  
 0   Well              36 non-null     object 
 1   Age_BA            36 non-null     float64
 2   Present_Depth_km  36 non-null     float64
 3   TOC_%             36 non-null     float64
 4   Requ_%            36 non-null     float64
 5   Quartz_%          36 non-null     float64
 6   Clay_%            36 non-null     float64
 7   Carbonate_%       36 non-null     float64
dtypes: float64(7), object(1)
memory usage: 2.4+ KB
```

In [6]:

```
MarcellusS1prediction.describe()
```

Out[6]:

	Age_BA	Present_Depth_km	TOC_%	Requ_%	Quartz_%	Clay_%	Carbonate
**count**	3.600000e+01	36.000000	36.000000	36.000000	36.000000	36.000000	36.000(
**mean**	3.900000e-01	2.139722	5.118611	1.661389	34.147222	50.216667	6.144
**std**	1.688957e-16	0.411801	3.002091	0.633206	8.285012	12.843486	5.339(
**min**	3.900000e-01	1.530000	1.940000	1.000000	19.600000	22.300000	1.000(
**25%**	3.900000e-01	2.000000	3.005000	1.270000	28.375000	40.500000	2.825(
**50%**	3.900000e-01	2.175000	4.090000	1.370000	32.350000	50.900000	4.200(
**75%**	3.900000e-01	2.350000	6.492500	2.100000	38.000000	60.000000	8.000(
**max**	3.900000e-01	2.840000	13.600000	2.680000	60.000000	71.000000	23.000(

In [7]:

```
X_new = MarcellusS1prediction.iloc[:, 1:].values
```

In [8]:

```
X_new
```

Out[8]:

```
array([[ 0.39,  1.53,  2.3 ,  1.  , 26.8 , 42.4 , 19.7 ],
       [ 0.39,  1.53,  5.1 ,  1.  , 24.8 , 52.4 ,  2.6 ],
       [ 0.39,  1.54,  6.  ,  1.  , 22.8 , 41.9 , 12.  ],
       [ 0.39,  1.54,  3.2 ,  1.  , 27.9 , 49.8 ,  4.4 ],
       [ 0.39,  1.54,  3.8 ,  1.  , 30.1 , 48.9 ,  2.9 ],
       [ 0.39,  1.54,  8.1 ,  1.  , 28.5 , 38.  ,  4.9 ],
       [ 0.39,  1.54, 10.2 ,  1.  , 30.7 , 34.1 ,  2.4 ],
       [ 0.39,  1.55, 12.8 ,  1.  , 23.1 , 31.  ,  6.2 ],
       [ 0.39,  1.55, 13.6 ,  1.  , 19.6 , 30.3 , 10.6 ],
       [ 0.39,  2.82,  3.4 ,  2.  , 33.6 , 44.9 ,  3.4 ],
       [ 0.39,  2.82,  3.1 ,  2.  , 35.4 , 40.7 ,  8.1 ],
       [ 0.39,  2.83,  6.4 ,  2.  , 32.7 , 39.9 ,  3.6 ],
       [ 0.39,  2.83,  8.3 ,  2.1 , 30.9 , 35.2 ,  7.4 ],
       [ 0.39,  2.84, 10.7 ,  2.1 , 44.4 , 22.3 ,  6.  ],
       [ 0.39,  2.17,  4.05,  1.36, 31.  , 65.  ,  4.  ],
       [ 0.39,  2.18,  3.33,  1.36, 37.  , 60.  ,  3.  ],
       [ 0.39,  2.18,  2.31,  1.36, 36.  , 63.  ,  1.  ],
       [ 0.39,  2.18,  4.28,  1.36, 32.  , 49.  , 19.  ],
       [ 0.39,  2.18,  4.53,  1.37, 32.  , 57.  , 11.  ],
       [ 0.39,  2.2 ,  6.22,  1.37, 42.  , 56.  ,  2.  ],
       [ 0.39,  2.2 ,  6.77,  1.37, 44.  , 33.  , 23.  ],
       [ 0.39,  2.33,  1.94,  1.37, 36.  , 64.  ,  1.  ],
       [ 0.39,  2.34,  3.02,  1.37, 30.  , 69.  ,  1.  ],
       [ 0.39,  2.35,  2.64,  1.37, 32.  , 67.  ,  1.  ],
       [ 0.39,  2.35,  5.66,  1.37, 60.  , 38.  ,  3.  ],
       [ 0.39,  2.36,  6.83,  1.38, 50.  , 44.  ,  6.  ],
       [ 0.39,  2.36,  4.81,  1.4 , 38.  , 60.  ,  2.  ],
       [ 0.39,  2.36,  4.13,  1.41, 45.  , 46.  ,  8.  ],
       [ 0.39,  2.15,  2.15,  2.67, 26.  , 71.  ,  3.  ],
       [ 0.39,  2.16,  2.71,  2.67, 26.  , 66.  ,  8.  ],
       [ 0.39,  2.16,  2.67,  2.67, 28.  , 69.  ,  3.  ],
       [ 0.39,  2.16,  2.96,  2.67, 44.  , 54.  ,  2.  ],
       [ 0.39,  2.16,  3.1 ,  2.67, 39.  , 52.  ,  8.  ],
       [ 0.39,  2.16,  7.28,  2.68, 36.  , 60.  ,  4.  ],
       [ 0.39,  2.17,  3.07,  2.68, 38.  , 53.  ,  9.  ],
       [ 0.39,  2.17,  2.81,  2.68, 36.  , 60.  ,  5.  ]])
```

In [9]:

```
from sklearn.preprocessing import StandardScaler
sc = StandardScaler()
X_new = sc.fit_transform(X_new)
```

In [10]:

```
X_new
```

Out[10]:

```
array([[-1.        , -1.5016269 , -0.95220083, -1.05932523, -0.89938838,
        -0.6172426 ,  2.57465341],
       [-1.        , -1.5016269 , -0.00628732, -1.05932523, -1.14421244,
         0.17240678, -0.67320856],
       [-1.        , -1.47699885,  0.29775631, -1.05932523, -1.3890365 ,
        -0.65672507,  1.11216586],
       [-1.        , -1.47699885, -0.64815721, -1.05932523, -0.76473515,
        -0.03290206, -0.33132835],
       [-1.        , -1.47699885, -0.44546145, -1.05932523, -0.49542868,
        -0.1039705 , -0.61622852],
       [-1.        , -1.47699885,  1.00719144, -1.05932523, -0.69128793,
        -0.96468832, -0.23636162],
       [-1.        , -1.47699885,  1.71662658, -1.05932523, -0.42198147,
        -1.27265158, -0.71119524],
       [-1.        , -1.4523708 ,  2.59497484, -1.05932523, -1.35231289,
        -1.51744289,  0.01055186],
       [-1.        , -1.4523708 ,  2.86523584, -1.05932523, -1.78075499,
        -1.57271835,  0.84625903],
       [-1.        ,  1.67539147, -0.58059195,  0.54234248, -0.06698658,
        -0.41983025, -0.5212618 ],
       [-1.        ,  1.67539147, -0.68193983,  0.54234248,  0.15335507,
        -0.75148299,  0.37142541],
       [-1.        ,  1.70001951,  0.43288681,  0.54234248, -0.17715741,
        -0.81465494, -0.48327511],
       [-1.        ,  1.70001951,  1.07475669,  0.70250925, -0.39749906,
        -1.18579015,  0.238472  ],
       [-1.        ,  1.72464756,  1.8855397 ,  0.70250925,  1.25506333,
        -2.20443785, -0.02743483],
       [-1.        ,  0.07456826, -0.36100489, -0.48272485, -0.38525786,
         1.167365  , -0.40730173],
       [-1.        ,  0.09919631, -0.60423979, -0.48272485,  0.34921432,
         0.77254031, -0.59723518],
       [-1.        ,  0.09919631, -0.94882257, -0.48272485,  0.22680229,
         1.00943512, -0.97710207],
       [-1.        ,  0.09919631, -0.28330485, -0.48272485, -0.26284583,
        -0.09607401,  2.4417    ],
       [-1.        ,  0.09919631, -0.19884829, -0.46670817, -0.26284583,
         0.5356455 ,  0.92223241],
       [-1.        ,  0.14845241,  0.37207808, -0.46670817,  0.96127446,
         0.45668056, -0.78716862],
       [-1.        ,  0.14845241,  0.55788252, -0.46670817,  1.20609852,
        -1.35951301,  3.20143379],
       [-1.        ,  0.46861705, -1.07381829, -0.46670817,  0.22680229,
         1.08840006, -0.97710207],
       [-1.        ,  0.4932451 , -0.70896593, -0.46670817, -0.50766989,
         1.48322475, -0.97710207],
       [-1.        ,  0.51787315, -0.83733991, -0.46670817, -0.26284583,
         1.32529487, -0.97710207],
       [-1.        ,  0.51787315,  0.18289538, -0.46670817,  3.16469098,
        -0.96468832, -0.59723518],
       [-1.        ,  0.5425012 ,  0.5781521 , -0.4506915 ,  1.94057069,
        -0.4908987 , -0.02743483],
       [-1.        ,  0.5425012 , -0.10425694, -0.41865814,  0.47162635,
         0.77254031, -0.78716862],
       [-1.        ,  0.5425012 , -0.33397879, -0.40264147,  1.32851055,
```

```
        -0.33296882,  0.35243206],
       [-1.        ,  0.02531216, -1.00287477,  1.61545985, -0.997318  ,
         1.64115463, -0.59723518],
       [-1.        ,  0.04994021, -0.81369207,  1.61545985, -0.997318  ,
         1.24632994,  0.35243206],
       [-1.        ,  0.04994021, -0.82720512,  1.61545985, -0.75249395,
         1.48322475, -0.59723518],
       [-1.        ,  0.04994021, -0.72923551,  1.61545985,  1.20609852,
         0.29875068, -0.78716862],
       [-1.        ,  0.04994021, -0.68193983,  1.61545985,  0.59403837,
         0.14082081,  0.35243206],
       [-1.        ,  0.04994021,  0.73017391,  1.63147653,  0.22680229,
         0.77254031, -0.40730173],
       [-1.        ,  0.07456826, -0.69207462,  1.63147653,  0.47162635,
         0.21978574,  0.54236551],
       [-1.        ,  0.07456826, -0.77990944,  1.63147653,  0.22680229,
         0.77254031, -0.21736828]])
```

In [11]:

```
print(model.predict(X_new))
```

```
[[2.9845467 ]
 [3.8604107 ]
 [3.3182776 ]
 [3.195042  ]
 [3.0421793 ]
 [2.5373392 ]
 [2.5453644 ]
 [2.5044882 ]
 [3.4281745 ]
 [3.0143514 ]
 [1.67386   ]
 [5.3681893 ]
 [3.7243223 ]
 [4.776375  ]
 [2.7303286 ]
 [1.5210965 ]
 [1.453183  ]
 [2.315579  ]
 [2.411799  ]
 [illegible]
```

In [12]:

```
from tensorflow.keras.models import save_model, load_model
import pandas as pd
```

In [13]:

```
model = load_model('./saved_models/my_tf_model',
 custom_objects=None,
 compile=True)
```

In [14]:

```
raw_dataset=pd.read_csv("BakkenS1prediction.csv",sep=",")
```

In [15]:

```
BakkenS1prediction= raw_dataset.copy()
BakkenS1prediction.head()
```

Out[15]:

	Well	Age_BA	Present_Depth_km	TOC_%	Requ_%	Quartz_%	Clay_%	Car
**0**	Well_1_Upper_Bakken	0.35	3.17	13.57	0.94	39.57	44.87	
**1**	Well_1_Upper_Bakken	0.35	3.18	7.41	0.94	45.08	42.29	
**2**	Well_1_Upper_Bakken	0.35	3.18	9.14	0.94	48.90	36.24	
**3**	Well_1_Upper_Bakken	0.35	3.18	13.33	0.94	63.59	24.07	
**4**	Well_1_Lower_Bakken	0.35	3.21	6.58	0.94	38.36	49.23	

In [16]:

```
BakkenS1prediction.info()
```

```
<class 'pandas.core.frame.DataFrame'>
RangeIndex: 16 entries, 0 to 15
Data columns (total 8 columns):
 #   Column            Non-Null Count  Dtype  
---  ------            --------------  -----  
 0   Well              16 non-null     object 
 1   Age_BA            16 non-null     float64
 2   Present_Depth_km  16 non-null     float64
 3   TOC_%             16 non-null     float64
 4   Requ_%            16 non-null     float64
 5   Quartz_%          16 non-null     float64
 6   Clay_%            16 non-null     float64
 7   Carbonate_%       16 non-null     float64
dtypes: float64(7), object(1)
memory usage: 1.1+ KB
```

In [17]:

```
BakkenS1prediction.describe()
```

Out[17]:

	Age_BA	Present_Depth_km	TOC_%	Requ_%	Quartz_%	Clay_%	Carbonate
**count**	1.600000e+01	16.000000	16.000000	16.000000	16.000000	16.000000	16.000
**mean**	3.500000e-01	3.080625	11.439375	0.932500	45.746875	42.092500	1.0987
**std**	5.733167e-17	0.222874	3.434986	0.061698	8.640546	9.339484	0.461
**min**	3.500000e-01	2.560000	6.580000	0.820000	38.040000	24.070000	0.150
**25%**	3.500000e-01	3.010000	8.295000	0.937500	38.420000	36.532500	0.975
**50%**	3.500000e-01	3.180000	11.885000	0.940000	43.315000	43.580000	1.250
**75%**	3.500000e-01	3.220000	13.570000	0.940000	50.270000	49.230000	1.425
**max**	3.500000e-01	3.270000	16.690000	1.110000	63.590000	53.920000	1.600

In [18]:

```
X_new = BakkenS1prediction.iloc[:, 1:].values
```

In [19]:

```
X_new
```

Out[19]:

```
array([[ 0.35,  3.17, 13.57,  0.94, 39.57, 44.87,  1.6 ],
       [ 0.35,  3.18,  7.41,  0.94, 45.08, 42.29,  1.34],
       [ 0.35,  3.18,  9.14,  0.94, 48.9 , 36.24,  0.6 ],
       [ 0.35,  3.18, 13.33,  0.94, 63.59, 24.07,  0.9 ],
       [ 0.35,  3.21,  6.58,  0.94, 38.36, 49.23,  1.5 ],
       [ 0.35,  3.22, 11.34,  0.94, 38.04, 53.92,  1.  ],
       [ 0.35,  3.22, 15.86,  0.94, 50.27, 36.63,  1.2 ],
       [ 0.35,  3.22,  8.59,  0.94, 38.44, 50.59,  0.15],
       [ 0.35,  3.27, 13.57,  0.94, 39.57, 44.87,  1.6 ],
       [ 0.35,  3.27,  7.41,  0.94, 45.08, 42.29,  1.34],
       [ 0.35,  2.56, 12.43,  0.86, 53.24, 35.41,  0.15],
       [ 0.35,  2.56, 13.33,  0.93, 63.59, 24.07,  1.  ],
       [ 0.35,  3.01,  6.58,  0.82, 38.36, 49.23,  1.5 ],
       [ 0.35,  3.01, 11.34,  0.85, 38.04, 53.92,  1.  ],
       [ 0.35,  3.01, 16.69,  0.95, 41.55, 49.22,  1.4 ],
       [ 0.35,  3.02, 15.86,  1.11, 50.27, 36.63,  1.3 ]])
```

In [20]:

```
from sklearn.preprocessing import StandardScaler
sc = StandardScaler()
X_new = sc.fit_transform(X_new)
```

In [21]:

```
X_new
```

Out[21]:

```
array([[ 0.        ,  0.41416232,  0.64061405,  0.12554609, -0.7383155 ,
         0.30714647,  1.12079843],
       [ 0.        ,  0.46050216, -1.21151034,  0.12554609, -0.07971088,
         0.0218403 ,  0.53943665],
       [ 0.        ,  0.46050216, -0.69135203,  0.12554609,  0.37688978,
        -0.64719162, -1.11520841],
       [ 0.        ,  0.46050216,  0.56845336,  0.12554609,  2.13277033,
        -1.99299634, -0.44440636],
       [ 0.        ,  0.59952168, -1.46106606,  0.12554609, -0.88294555,
         0.78929179,  0.89719774],
       [ 0.        ,  0.64586152, -0.02987904,  0.12554609, -0.92119482,
         1.30792976, -0.22080568],
       [ 0.        ,  0.64586152,  1.3291473 ,  0.12554609,  0.54064447,
        -0.60406395,  0.22639569],
       [ 0.        ,  0.64586152, -0.85672028,  0.12554609, -0.87338323,
         0.93968575, -2.12141149],
       [ 0.        ,  0.87756072,  0.64061405,  0.12554609, -0.7383155 ,
         0.30714647,  1.12079843],
       [ 0.        ,  0.87756072, -1.21151034,  0.12554609, -0.07971088,
         0.0218403 ,  0.53943665],
       [ 0.        , -2.41256793,  0.29785077, -1.21361223,  0.8956455 ,
        -0.73897617, -2.12141149],
       [ 0.        , -2.41256793,  0.56845336, -0.0418487 ,  2.13277033,
        -1.99299634, -0.22080568],
       [ 0.        , -0.32727512, -1.46106606, -1.88319139, -0.88294555,
         0.78929179,  0.89719774],
       [ 0.        , -0.32727512, -0.02987904, -1.38100702, -0.92119482,
         1.30792976, -0.22080568],
       [ 0.        , -0.32727512,  1.57870302,  0.29294088, -0.50164814,
         0.78818596,  0.67359706],
       [ 0.        , -0.28093528,  1.3291473 ,  2.97125752,  0.54064447,
        -0.60406395,  0.44999638]])
```

In [22]:

```
print(model.predict(X_new))
```

```
[[5.9196873]
 [2.0221586]
 [1.3083813]
 [3.3798652]
 [2.4263053]
 [4.4843583]
 [4.5060062]
 [1.8996017]
 [5.3731914]
 [1.9284317]
 [3.0873027]
 [2.624426 ]
 [3.9669528]
 [6.004355 ]
 [7.6387672]
 [1.7363921]]
```

In [ ]:

```

```
